# Microbial Community Analysis of Colored Snow from an Alpine Snowfield in Northern Japan Reveals the Prevalence of *Betaproteobacteria* with Snow Algae

**DOI:** 10.3389/fmicb.2017.01481

**Published:** 2017-08-07

**Authors:** Mia Terashima, Kazuhiro Umezawa, Shoichi Mori, Hisaya Kojima, Manabu Fukui

**Affiliations:** Institute of Low Temperature Science, Hokkaido University Sapporo, Japan

**Keywords:** snow algae, snow microbial community, algae–bacteria interaction, *Betaproteobacteria*, *Chloromonas*, Japan

## Abstract

Psychrophilic algae blooms can be observed coloring the snow during the melt season in alpine snowfields. These algae are important primary producers on the snow surface environment, supporting the microbial community that coexists with algae, which includes heterotrophic bacteria and fungi. In this study, we analyzed the microbial community of green and red-colored snow containing algae from Mount Asahi, Japan. We found that *Chloromonas* spp. are the dominant algae in all samples analyzed, and *Chlamydomonas* is the second-most abundant genus in the red snow. For the bacterial community profile, species belonging to the subphylum *Betaproteobacteria* were frequently detected in both green and red snow, while members of the phylum *Bacteroidetes* were also prominent in red snow. Furthermore, multiple independently obtained strains of *Chloromonas* sp. from inoculates of red snow resulted in the growth of *Betaproteobacteria* with the alga and the presence of bacteria appears to support growth of the xenic algal cultures under laboratory conditions. The dominance of *Betaproteobacteria* in algae-containing snow in combination with the detection of *Chloromonas* sp. with *Betaproteobacteria* strains suggest that these bacteria can utilize the available carbon source in algae-rich environments and may in turn promote algal growth.

## Introduction

During the snowmelt season in the spring and summer months, algal blooms appear on alpine snowfields, coloring the snow green and red. These psychrophilic algae make use of the window of opportunity where conditions are sufficient for growth, followed by mating and returning to a resting state to survive extreme conditions such as high light, freezing conditions and desiccation (Hoham and Duval, 2001). These types of colored snow have been observed in polar and alpine environments, and the majority of the blooms are caused by green algae from the order *Chlamydomonadales* (Remias et al., 2005, 2016). Snow algae have been ubiquitously found across the world ranging from Arctic and Antarctic regions (Newton, 1982; Ling and Seppelt, 1990), alpine regions of North America (Thomas and Duval, 1995), South America (Takeuchi and Kohshima, 2004), Australia (Marchant, 1982), New Zealand (Novis et al., 2008), North Africa (Duval et al., 1999), Europe (Remias et al., 2010), Siberia (Hisakawa et al., 2015; Tanaka et al., 2016), and Asia (Muramoto et al., 2010). Algal blooms have been shown to reduce glacial surface albedo, having a direct impact on glacial snow and ice melt rates (Duval et al., 1999; Yallop et al., 2012; Lutz et al., 2014, 2016). Furthermore, algae are the dominant primary producers in snow during melt season, supporting a diverse microbial community, including heterotrophic bacteria and fungi that depend on fixed carbon from algae, as well as invertebrates that act as consumers and predators (Williams et al., 2003; Mannisto and Haggblom, 2006; Hodson et al., 2010; Zawierucha et al., 2015; Hamilton and Havig, 2017). In addition to carbon fixation, snow algae have been shown to be key sequesters of nutrients such as iron, manganese, and phosphate (Hamilton and Havig, 2017). Nutrient cycling derived from bioactivity of algae and its associated microbial community is an important factor in snow ecosystem as well as downstream ecosystems affected by nutrients provided by the snowmelt (Boetius et al., 2015).

Along with green algae, other phototrophs, such as golden algae and cyanobacteria, and heterotrophic invertebrates, fungi and bacteria have been found in snow (Remias et al., 2013a; Maccario et al., 2014; Zawierucha et al., 2015). In addition to these organisms competing for nutrients in their shared environment, species can coexist in mutualistic (symbiotic and syntrophic) and parasitic relationships (Little et al., 2008; Konopka, 2009). Cross-feeding between phototrophs and heterotrophic bacteria have been widely reported (Seth and Taga, 2014). While some relationships appear to be totally commensal, where only the bacteria depend on the phototroph for fixed carbon, many have a mutualistic interaction, consisting of the phototrophic partner providing fixed carbon in exchange for another essential nutrient (such nitrogen, phosphorus, sulfur, and vitamins) (Croft et al., 2005; Ramanan et al., 2016). Additionally, some interactions start mutualistic and can turn parasitic, such as when the bacteria initially nurture growth and at a later stage kill the algae (Segev et al., 2016). Interactions between snow algae and bacteria are not well-characterized, but the abundance of bacteria in algal snow samples in addition to the observation of Gram-negative bacteria in the mucilage surrounding the cell wall of resting algal cells from independently obtained red snow samples suggest a possible specificity in these interactions (Weiss, 1983; Duval et al., 1999; Gawor et al., 2016).

Identification of bacteria found in snow containing snow algae have been performed on samples from the Arctic, Antarctica and North America, and Japan revealed a particularly high abundance of bacteria belonging to the phylum *Bacteroidetes* and subphylum *Betaproteobacteria* (Segawa et al., 2005; Fujii et al., 2010; Lutz et al., 2015a,b, 2017; Hamilton and Havig, 2017). In the current study, we aim to broaden the research by analyzing the microbial community associated with snow Algae from Hokkaido, Japan. Furthermore, identifying the bacterial community in both green and red snow will provide a more comprehensive overview of the bacteria present in algae-containing snow, as it has been reported that snow algae can shift between vegetative cells to cysts that often accumulate astaxanthin pigments (Remias et al., 2005). To this end, we isolated green and red snow from Mount Asahi, in Hokkaido, Japan, to identify the dominant algae and the bacterial community profiles. In addition to the analysis of environmental samples, we cultured algal strains from the snow samples to determine the presence of bacteria co-growing with the algae.

## Materials and Methods

### Field Site and Sample Collection

Snow samples were collected on June 15th (sampling day 1) and June 22nd 2016 (sampling day 2) from two locations near lake Sugatami on Mount Asahi in the Daisetsu National Park in Hokkaido, Japan (altitude, 1670 m; site A, N43°39′39.46″ E142°49′45.93″; site B, N43°39′40.29″ E142°49′59.90″). Red snow was observed in both sample sites, while green snow was only observed at site B on June 22nd. Letters A and B will be used to designate sampling location and numbers 1 and 2 will be used to designate each of the sampling day. Colored snow patches were always found adjacent to a steeper hill and appeared to emerge more strongly along grooves and water paths on the snow surface. Remaining snow depth at sampling site was ∼90 cm on June 15th and ∼50 cm on June 22nd. Both red and green coloration on the snow were observed on the top 3–5 cm of the snow. Both sampling sites are areas out of bounds to hikers and the top 5 cm of the snow that was visibly red or green colored was collected using a hand shovel into sterile sampling bags (1260 ml bags, Thermo Fisher Scientific). Samples were stored in cooler box on ice during transport back to the laboratory until further processing (∼4 h). 50 ml volume of snow was melted on site to determine the pH and conductivity using a hand-held meter (DKK-TOA EC/pH meter WM-32EP with electrical conductivity cell CT-27112B). Details of sample processing and preparation immediately after returning to the laboratory are described below under each relevant section. Small volumes (<50 ml) of snow for cell density, dissolved organic carbon (DOC), total ammonium, total phosphate and ion chromatograph analyses were melted immediately for processing. Larger volumes (1 L) for DNA extraction were melted overnight at 4°C. Samples for cell counting (15 ml melted snow sample) were fixed with 1% final concentration of glutaraldehyde and stored at 4°C. Cells were counted using a Thoma counter.

### Cell Culturing

Freshly melted samples were plated onto agar plates with Bold’s basal medium for algae with triple nitrogen concentration at pH 5.5 and at pH 7 (3NBBM5.5 and 3NBBM7) (Nichols and Bold, 1965). The medium was composed of: 8.7 mM NaNO_3_, 0.3 mM MgSO_4_⋅7H_2_O, 0.43 mM NaCl, 0.43 mM K_2_HPO_4_, 1.3 mM KH_2_PO_4_, 0.17 mM CaCl_2_⋅2H_2_O, 31 μM ZnSO_4_⋅7H_2_O, 7.3 μM MnCl_2_⋅4H_2_O, 4.9 μM MoO_3_, 6.3 μM CuSO_4_⋅5H_2_O, 1.7 μM Co(NO_3_)_2_⋅6H_2_O, 0.19 mM H_3_BO_3_, 0.17 mM EDTA⋅Na_2_, 0.55 mM KOH, 18 μM FeSO_4_⋅7H_2_O with 1.5% agar. 100 μl volumes of melted snow were plated out undiluted, and with 1:10 and 1:100 dilutions. For liquid culture experiments, Bold’s basal medium was prepared without agar. All cultures were grown at 5°C under 100 μmol photons m^-2^ s^-1^ white fluorescent light. For checking bacteria presence and growth in algal strains, cultures were grown in the dark in Reasoner’s 2A (R2A) medium (Daigo) at 5°C.

### Snow Geochemistry Analyses

For geochemical analysis of snow, 50 ml of melted snow samples were filtered using a 0.22 μm syringe filter and stored at -20°C. DOC was analyzed using a Shimadzu TOC-V/CSN with three replicates of 100 μl injection volume as described previously (Fujii et al., 2012). Total ammonia was measured using the Sigma ammonia assay kit (AA0100, detection limit of 11 μM) and total phosphate was measured using the Abcam colorimetric phosphate assay kit (ab65622, detection limit of 1 μM). A Dionex ICS-1500 ion chromatograph was used to measure chloride, nitrate, and sulfate.

### Chlorophyll Fluorescence Transient Measurement and Pigment Analysis

Chlorophyll *a* fluorescence kinetics were measured by monitoring the previously defined O, J, I, and P steps (referred to as “OJIP transient”) in chlorophyll fluorescence after excitation with saturating light at an intensity of 3,000 μmol photons m^-2^ s^-1^ (Strasser et al., 2000; Boisvert et al., 2006). OJIP test was performed using the 50 ml volumes of green snow B2 and red snow B2 samples that were melted overnight at 4°C. The cells were dark-adapted for 30 min and the OJIP was measured using an AquaPen-P (Photon Systems Instruments). For pigment analysis, a total of ∼3 × 10^5^ cells for each sample (3 ml of red snow B2 and 10 ml of green snow B2) were pelleted (8,000 g, 5 min), supernatant removed and flash-frozen in liquid nitrogen. Pigments were extracted with 300 μl of acetone and 10 μl of extracts were subjected to HPLC analysis using the Hitachi L-7000 HPLC system (Hitachi High-Technologies Co. Ltd., Tokyo, Japan) equipped with a photodiode array detector (L-2450; Hitachi). Pigments were separated on a C18 reverse phase column, YMC AL303 (250 mm × 4.6 mm; YMC Co. Ltd., Kyoto, Japan), at 40°C as described in Sarada et al. (2006). The mobile phase consists of solvent A (methanol:H_2_O = 9:1 [v/v]) and solvent B (acetone) with a flow rate of 1 ml min^-1^. The mobile phase was 80% A and 20% B at the time of injection, and solvent A concentration was linearly decreased to 20% over 25 min, after which the same concentration was kept for an additional 10 min to elute all esterified carotenoids. The eluate was monitored with the photodiode array detector in the range from 350 to 700 nm. Pigments were identified as described in Fujii et al. (2010).

### 16S and 18S rRNA Gene Sequencing

For cell collection for DNA extraction, ∼1 L volume of snow was melted overnight during the first night after sampling at 4°C. Once the snow melted, 150 ml volumes were passed through Sterivex filters (SVGPL10RC) to collect cells and these filters were stored at -20°C. DNA was extracted using the PowerWater sterivex DNA isolation Kit (Mo Bio Laboratories) following the manufacturer’s instructions, leading to a total of approximately 0.5–1 μg of DNA extracted per filter. 16S rRNA gene was amplified over the V3–V4 hypervariable region using the following primer pair 341F (5′ CCTACGGGNGGCWGCAG) and 805R (5′ GACTACHVGGGTATCTAATCC) (Herlemann et al., 2011; Klindworth et al., 2013). The 18S rRNA gene was amplified over the V4–V5 hypervariable region using the primer pair 528F (5′ GCGGTAATTCCAGCTCCAA) and 706R (5′ AATCCRAGAATTTCACCTCT) (Cheung et al., 2010; Lutz et al., 2015b). All primers were tagged with the Illumina Nextera Transposase adapter sequences on the 5′ end of the primers (forward adapter: 5′ TCGTCGGCAGCGTCAGATGTGTATAAGAGACAG; reverse adapter: 5′ GTCTCGTGGGCTCGGAGATGTGTATAAGAGACAG). For Polymerase Chain Reaction (PCR) gene amplification, KAPA HiFi HotStart ReadyMix polymerase was used with 12.5 ng of total DNA per reaction with 0.2 μM final concentration of each primer in a 25 μl reaction volume. For the 16S rRNA gene amplification, the initial denaturation was at 95°C for 5 min, followed by 25 cycles of 30 s at 95°C, 30 s at 50°C, 30 s at 72°C, with the final extension at 72°C for 5 min. 18S rRNA gene amplification was carried out as in Lutz et al. (2015b) with the initial denaturation at 95°C for 5 min, followed by 30 cycles of 30 s at 95°C, 30 s at 60°C, 30 s at 72°C, and the final extension at 72°C for 7 min. PCR products were purified using the QIAquick PCR purification kit (Qiagen). Purified PCR product was submitted for quality analysis and Illumina MiSeq sequencing at a sequencing facility (Hokkaido Systems Science Co., Ltd., Sapporo, Japan). QIIME was used to cleanup and assign operational taxonomic units (OTUs) from raw sequencing data (Caporaso et al., 2010). Sequence cleanup was performed by trimming sequences after an ambiguous base and when the quality score fell below 25 in a sliding window of 50 bp. Fastq-join was used with the default settings (minimum 6 bp overlap and an 8% maximum mismatch) (Aronesty, 2011). Sequences not in the 200–1000 bp range as well as sequences containing homopolymers (8 bp or longer) were removed. Sequences were filtered for the presence of the forward primers (1 mismatch allowed), followed by the trimming of the forward and reverse primers. OTUs were picked from the quality-filtered sequences using the UCLUST method with a similarity threshold of 97% (Edgar, 2010). For the 18S rRNA gene fragment analysis, at least 100 reads were required for the picked OTUs and sequences were aligned using SINA with the SILVA database (Pruesse et al., 2012). Default settings were used with the minimum identity threshold set to 0.9. For the 16S rRNA gene fragment analysis, the QIIME annotation program was used with the 16S rRNA gene database (greengenes version 13_8 database) with a minimum identity threshold of 0.9 (DeSantis et al., 2006). The representative 16S rRNA OTUs were subjected to chimera-checking using QIIME ChimeraSlayer program.

For sequencing of 18S rRNA gene of isolated *Chloromonas* strains, DNA was extracted from 1 ml of liquid culture using phenol-chloroform as described previously (Newman et al., 1990). Primer pair E4F (5′ CTGGTTGATTCTGCCAGT) and E1628R (5′ CGACGGGCGGTGTGTA) was used for PCR amplification of 18S rRNA gene (van Hannen et al., 1999; Kojima et al., 2009). Primers E4F, 528F, 706R, and 1628R were used for sequencing the gene fragment in addition to custom primers to read the gaps and ends of the amplicon: C1_F1 (5′ GACCGGAGTAATGATTAAGAG), C1_F2 (5′ ACTATTGTCGTTTAGGCAATG), C1_R1 (5′ GAATTACTACGGTTATCCGAG), and C1_R2 (5′ GGGCAGAAATTTGAATGAAAC).

The 16S and 18S rRNA gene fragment sequences obtained by MiSeq analysis have been submitted to NCBI Sequence Read Archive (SRA) database under the accession No. SRR5482949-SRR5482956. The 18S rRNA gene fragment of the *Chloromonas* sp. AsaC1 strain has been deposited in GenBank under the accession number KY993906.

### PCR-Denaturing Gradient Gel Electrophoresis Analysis

For PCR-Denaturing Gradient Gel Electrophoresis (DGGE) analysis, algal cultures were grown in 3NBBM liquid media (as described above) in 10 ml volumes for 3 weeks until around 0.5 million cells ml^-1^ concentration of algal cells. The cells were collected by centrifugation (8,000 *g*, 5 min) followed by phenol-chloroform extraction of DNA. A two-step nested-PCR approach was used to eliminate chloroplast and mitochondrial contamination. The first round of PCR was performed using bacterial-specific primer pair 63F (5′ GCAGGCCTAACACATGCAAGTC) and 1175R (5′ ACGTCRTCCMCACCTTCCTC) with the initial denaturation at 95°C for 5 min, followed by 25 cycles of 30 s at 95°C, 30 s at 53°C, 2 min at 72°C, and the final extension at 72°C for 7 min. (Dorn-In et al., 2015). The PCR product was gel extracted and purified (QIAquick Gel Extraction Kit, QIAGEN). 0.5 ng of DNA was used for the second round of PCR with primer pair GC341F and 907R as described previously (Muyzer et al., 1995). PCR and DGGE were performed exactly as described previously, except that the gel was stained with SYBR green for the visualization of bands (Fujii et al., 2010). 10 μl of PCR product was loaded on the DGGE gel for each sample. As described in Fujii et al. (2010) specific bands were excised from the DGGE gel and re-amplified using the same primers (GC341F and 907R) for sequencing.

## Results

### Environmental Parameters and Cell Densities

**FIGURE 1:**
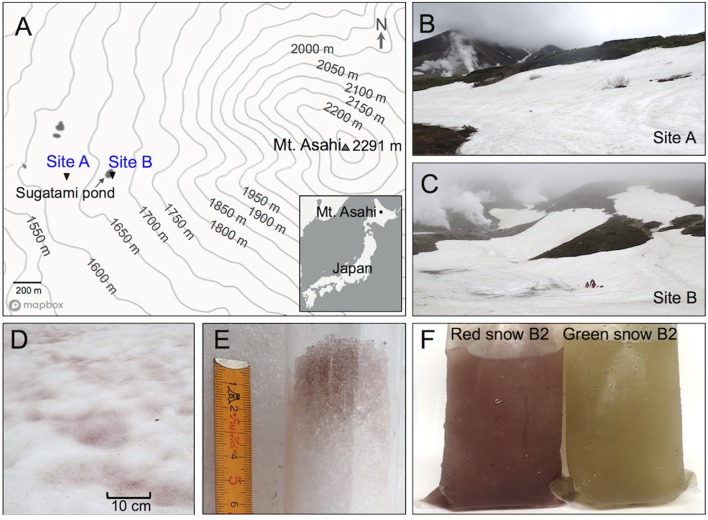
**(A)** Sampling sites of Mount Asahidake. **(B)** Sampling site A snowfield. **(C)** Sampling site B snowfield. **(D)** Red snow surface of sampling site A snowfield. **(E)** Removing a core from the snow (sample red snow B2) shows coloration from the algae in the top 3 cm of the snow. **(F)** Melted green and red snow samples from B2 sampling. (Map generated by © Mapbox, © OpenStreetMap, modified and annotated).

Red snow was observed during the snowmelt season of June to July, 2016, on the snowfields of Mount Asahi, on Hokkaido, the northern island of Japan. Mount Asahi is an active stratovolcano and red snow was found in patches on snowfields that were flat or at a slight slope, often adjacent to higher, more exposed grounds that had faster snowmelt (**Figure 1**). The color was very light pink with some darker areas, in grooves or melted ridges or dents on the snow surface. Two sites, designated A and B, were chosen for sampling on two dates (day 1: June 15th; day 2: 22nd, 2016). Green snow was much more elusive and was only found at sampling site B on June 22nd, 2016. The green and red snow at sampling site B were only around 3 m apart from one another. Colored snow was present at the top ∼3 cm of the snow surface (**Figure 1**). Cell density analysis revealed a lower density for the green snow (∼3 × 10^4^ cells ml^-1^) compared to the red snow (∼ 1 × 10^5^ cells ml^-1^). On the snow surface, the green coloration was very pale and difficult to see compared to the red coloration, but melted snow directly showed a stark difference in coloration (**Figure 1F**).

The pH-values were very similar between all samples (**Table 1**). The conductivity of the snow samples was 10 μS cm^-1^ or below. DOC and major ions were measured for all samples as well as for white snow at site B for comparison (**Table 1**). DOC levels in algae-containing snow were three–eightfold higher compared to white snow. Phosphate and ammonia concentrations were below detection levels for all samples, aside from ammonia in green snow at 66.5 μM. Nitrate concentrations ranged from 2.4 to 3.6 μM and chloride concentrations ranged from 9.4 to 17.4 μM for all algae-containing samples. Sulfate concentration was over threefold higher in the green snow at 21.9 μM compared to the red snow samples (2.0–3.2 μM).

**Table 1:** Cell density, pH, conductivity, and concentrations of aqueous dissolved carbon (DOC) and major ions in the filtered snow samples.

	Red snow A1	Red snow B2	Green snow B2	White snow B2
Cell density	∼1 × 10^5^ cells ml^-1^	∼1 × 10^5^ cells ml^-1^	∼3 × 10^4^ cells ml^-1^	<100 cells ml^-1^
pH	5.61	5.48	5.44	5.58
Conductivity	<10 μS cm^-1^	10 μS cm^-1^	10 μS cm^-1^	<10 μS cm^-1^
DOC	162 μM	424 μM	268 μM	54 μM
PO_4_^3-^	bdl (<1 μM)	bdl (<1 μM)	bdl (<1 μM)	bdl (<1 μM)
NH_4_^+^	bdl (<11 μM)	bdl (<11 μM)	66.5 μM	bdl (<11 μM)
NO_3_^-^	3.0 μM	3.6 μM	2.4 μM	1.6 μM
Cl^-^	14.4 μM	9.4 μM	17.4 μM	6.0 μM
SO_4_^2-^	2.0 μM	3.2 μM	21.9 μM	1.3 μM


*bdl, below detection limits.*

### Green and Red Snow Samples Are Distinct in Cell Morphology, Pigment Composition and Photosynthetic Activity

**FIGURE 2:**
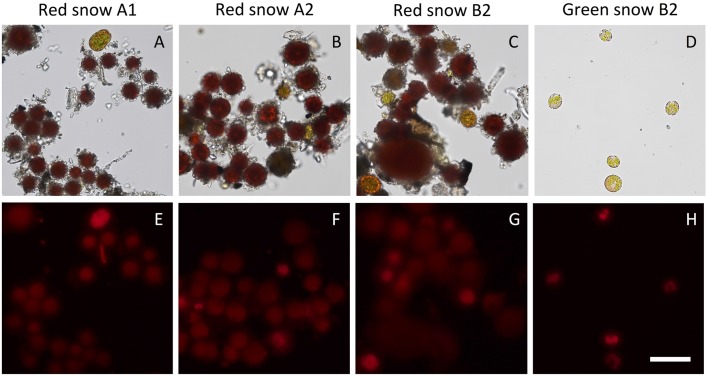
Microscopy images of three red snow samples and one green snow sample from Mt. Asahi. **(A–D)** Brightfield images, **(E–H)** chlorophyll autofluorescence. Scale bar represents 50 μm for all images.

Observation of melted green and red snow samples under the microscope revealed that the algal cells in the green snow are very homogeneous: all are around 10–20 μm in size, spherical and non-aggregating (**Figure 2D**). Red snow consisted of algal cells that were varying in size and coloration (**Figures 2A–C**). Most cells were between 10 and 30 μm, although there were some very large cells that were over 50 μm in diameter. The majority of the cells showed red coloration, and there were also few that were orange and green. Chlorophyll autofluorescence was visibly stronger in the green-colored cells, whereas the red-colored cells showed weak fluorescence, which could be due to debris attached to the cell surface (**Figures 2E–H**). Consistent to the fluorescence observed by microscopy, OJIP transient chlorophyll fluorescence suggested stronger photosynthetic activity for the green snow (Supplementary Figure S1). HPLC pigment analysis of equal cell counts of each sample detected chlorophyll in green snow and red snow samples, while prominent peaks from astaxanthin pigments were detected from the red snow, but not from green snow (Supplementary Figure S2).

### 18S rRNA Gene Sequencing Identified *Chloromonas* spp. As Prevalent in Both Green and Red Snow

**FIGURE 3:**
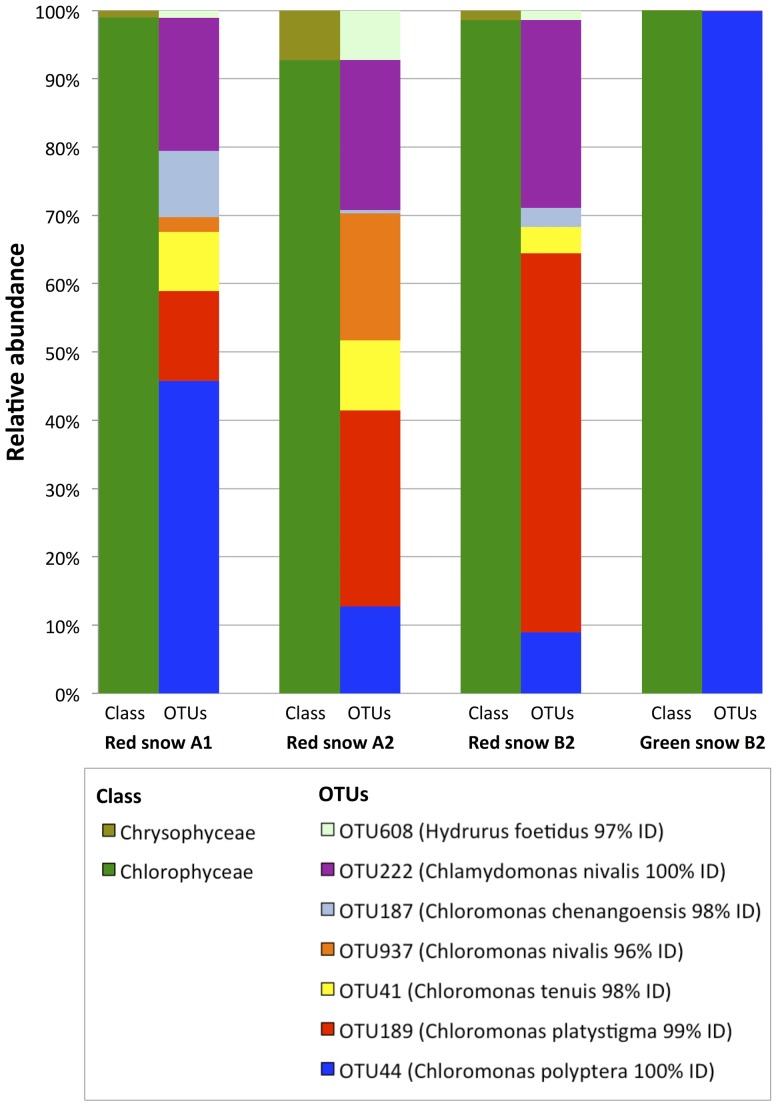
Algal community composition based on 18S rRNA gene sequencing of three red snow samples (red snow A1, A2, and B2) and one green snow sample (green snow B2) from Mt. Asahi. Left bar represents the community composition at the class level and the right bar represents the individual OTUs (based on 97% similarity clustering) that make up the community.

Sequencing results indicated that algae belonging to the class *Chlorophyceae* were the most dominant in all snow samples (**Figure 3**). Sequences belonging to the *Chloromonas* genus were the most abundant, with the majority (99%) of the green snow algal reads were from OTU44, which BLAST-aligned identically to *Chloromonas polyptera* (Supplementary Table S1). For the red snow, OTU44 was also abundant, along with OTU189, which showed high similarity to *C. platystigma*. Among all algal reads combined, OTU222 was the third most abundant and aligned to *Chlamydomonas*. Aside from these highly abundant OTUs belonging to the *Chlamydomonadaceae* family, other algal species were also identified belonging to the *Chrysophyceae* family (OTU608 and OTU512). However, these sequences were detected less than 10-fold lower in frequency compared to the most highly detected *Chloromonas* sequence in each sample. These sequencing profiles indicate that the green and red coloration observed in the snow of Mount Asahi during our sampling dates are largely due to the high abundance of algae belonging to the *Chlamydomonadaceae* family, of which species belonging to the *Chloromonas* and *Chlamydomonas* genera were the most dominant.

**FIGURE 4:**
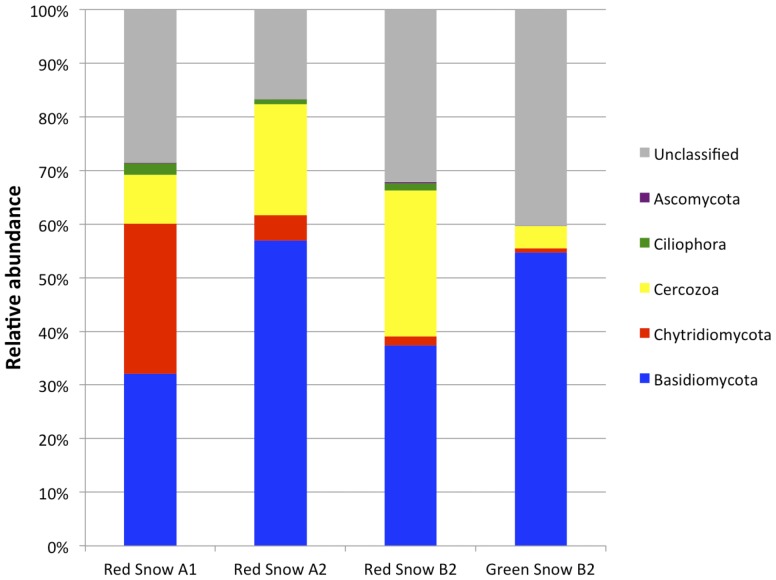
Non-algal community composition based on 18S rRNA gene sequencing of three red snow samples (red snow A1, A2, and B2) and one green snow sample (green snow B2) from Mt. Asahi. The community composition is represented by grouping the OTUs at the phylum level.

Aside from algal species, 18S rRNA gene sequencing identified other eukaryotic organisms (**Figure 4**). All snow samples had high sequencing reads for phyla *Basidiomycota*, *Cercozoa*, and *Chytridiomycota*, with *Basidiomycota* fungus *Phenoliferia psychrophila* (OTU43) having the highest read (Supplementary Table S2). Unclassified organisms made up a large fraction of the reads, with OTU156 being the most prominent, with a low similarity to the top BLAST hit, which is a single-celled eukaryote *Cercozoa* sp.

### 16S rRNA Gene Sequencing Detected *Betaproteobacteria* Abundantly in all Samples, with *Bacteroidetes* Also Prevalent in Red Snow Samples

**FIGURE 5:**
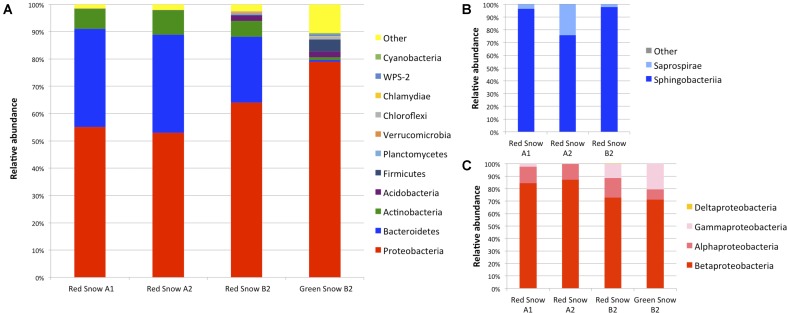
Bacterial community composition based on 16S rRNA gene sequencing of three red snow samples (red snow A1, A2, and B2) and one green snow sample (green snow B2) from Mt. Asahi. **(A)** Community composition of the OTUs grouped at the phylum level. **(B)** Composition of the *Bacteroidetes* phylum for the red snow samples. **(C)** Composition of the *Proteobacteria* phylum for the red and green snow samples.

Among the red snow samples, the dominant phyla were unchanged despite the different collection dates and location, with *Proteobacteria* and *Bacteroidetes* being the most prominent, together making up of around 90% of the total reads (**Figure 5**). Members of *Proteobacteria* consisted of around 53–64% of the reads in the red snow samples while members of *Bacteroidetes* consisted of between 24 and 36%. Bacteria belonging to the phylum *Actinobacteria* were the next most abundant with less than 10% of the total reads. In the green snow sample, *Proteobacteria* were detected frequently, but contrary to the red snow, bacteria in the phylum *Bacteroidetes* were detected in only trace levels.

Within *Proteobacteria*, members of the subphylum *Betaproteobacteria* were the most abundant with the majority of the sequencing reads stemming from six OTUs (OTU3276, 4031, 1414, 1284, 312, 3245) (Supplementary Table S3). All of these OTUs classified to the order *Burkholderiales* and BLAST analysis indicated that they belong to the genera *Actimicrobium*, *Herminiimonas*, *Glaciimonas*, *Aquaspirillum*, and *Polaromonas*. For members of the phylum *Bacteroidetes*, detected in the red snow samples, the sequences were much less diverse and ∼80% or more of the reads for each sample stemmed from OTU3271, which is classified to the family *Sphingobacteriaceae* (Supplementary Table S4). This bacterium shows no sequence similarities to characterized bacterial strains, as the closest BLAST hit was to *Solitalea koreensis* with only 91% sequence identity. However, the strain does appear to show similarities to other uncultured strains identified from glacier samples.

### Laboratory Growth of Environmental Isolates of *Chloromonas* sp. Co-culture with *Betaproteobacteria*

In order to isolate green algal strains, melted snow was immediately plated onto minimal medium plates at pH 5.5 and 7.0 (3NBBM plates). Green snow samples yielded no algal colonies, red snow yielded ∼40 colonies/100 μl of snow at pH5.5, whereas around 10-fold less algal colonies grew on plates at pH 7.0. Around 20 colonies were picked and seven strains (AsaC1-C7) could be maintained over several propagations. From these strains, 18S rRNA gene partial sequencing revealed all to be an exact match to *Chloromonas* sp. OTU189 that has a high similarity to *C. platystigma*. 1623 bp of 18S rRNA gene was sequenced for colony 1 (designated *Chloromonas* sp. AsaC1), which had the highest similarity (99% identity) to *C. platystigma* strain CCCryo 020-99 (NCBI accession: AF514401).

**FIGURE 6:**
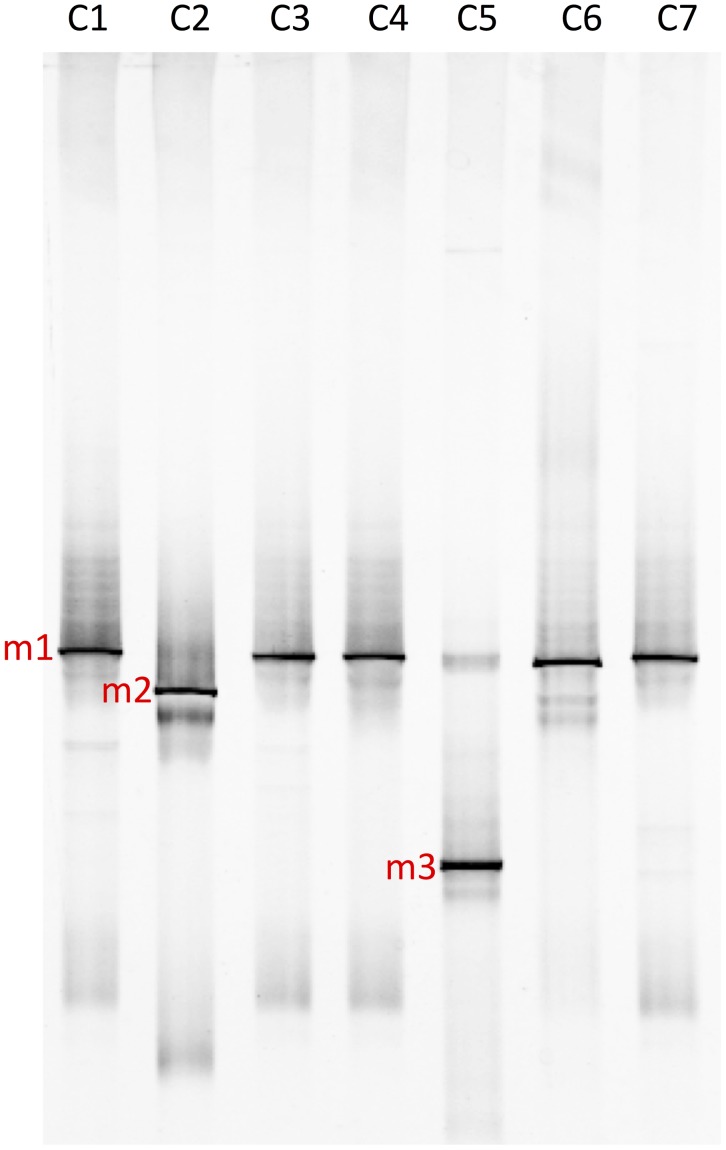
Polymerase Chain Reaction-DGGE profile of 16S rRNA gene fragments. Bands marked on the gel were excised, PCR-amplified and identified via sequencing. Major bacterial bands (marked m1–m3) are colored in red. BLAST top hit of sequencing results suggested that the bands are closely related to the following genera (all with 99% sequence identity): m1, *Glaciimonas* sp.; m2, *Polaromonas* sp.; and m3, *Actinomycetales* bacterium.

Denaturing Gradient Gel Electrophoresis analysis indicated that all of the independently isolated and cultured strains of *Chloromonas* sp. AsaC1-C7 contained one or more *Betaproteobacteria* in the *Burkholderiales* order (**Figure 6**). Members of the *Bacteroidetes* phylum were not detected, despite the fact that all strains were isolated from red snow, which had a high abundance of bacteria belonging to this phylum. Algal isolates AsaC1 and AsaC3-C7 contained band m1, which is related to *Glaciimonas* sp. (NCBI accession: AB991649), and band m2 was found in algal isolates AsaC2 and is related to *Polaromonas* sp. (NCBI accession: JX949585). Band m1 matched to OTU1414 (>98% identity) and band m2 matched to OTU312 (>98% identity), both abundantly found in the sequencing results (Supplementary Table S3). Band m3 was found in algal isolate AsaC5 and is related to *Actinomycetales* bacterium (GenBank: JX491372). Other minor bands from the DGGE were likely artifacts from PCR or heteroduplex bands, as all of them were identical in sequence to either m1 or m2 (**Figure 6**) (Neilson et al., 2013).

**FIGURE 7:**
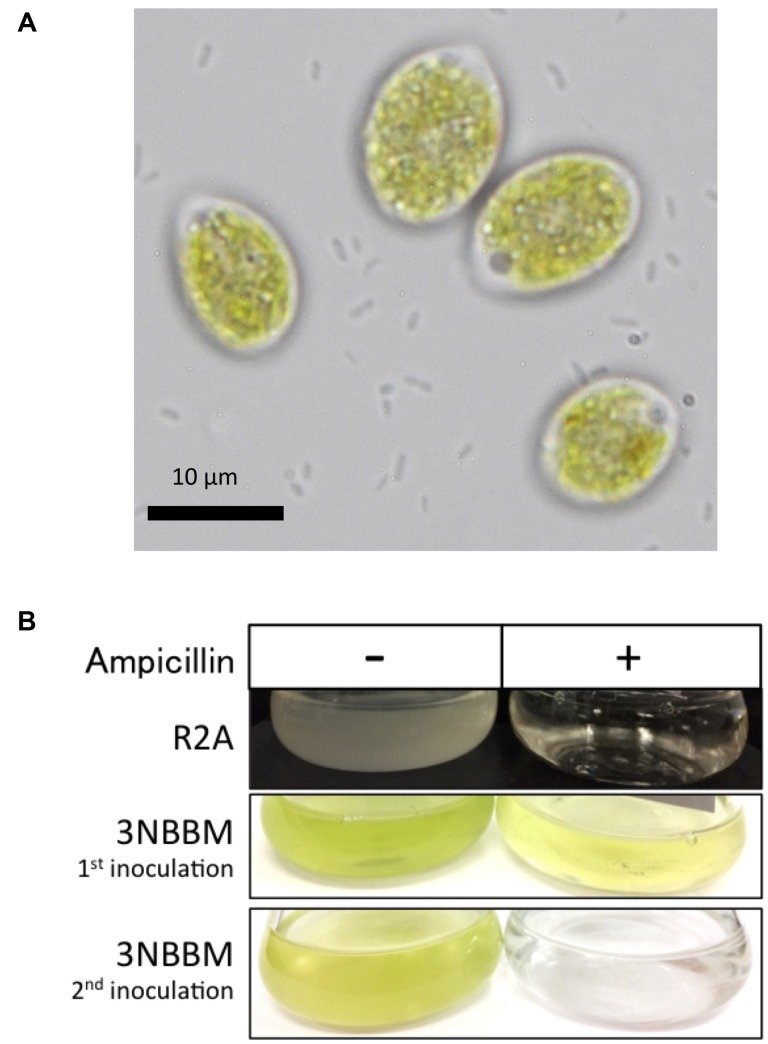
Bacteria co-isolate with *Chloromonas* sp. AsaC1. **(A)** Microscopy shows the presence of free-living bacteria with the algae culture. Scale bar represents 10 μm. **(B)** Bacteria can be cultured without the presence of algae in the dark in R2A media, but is unable to grow under 100 μg ml^-1^ ampicillin treatment. Elimination of bacteria with ampicillin hinders sustained algal growth.

Further analysis was performed with the isolated strain *Chloromonas* sp. AsaC1 and microscopy revealed free-living bacteria present in the algal culture (**Figure 7A**). Treatment with ampicillin to eliminate bacteria from the algal cultures prevented sustained growth of the algal cultures (**Figure 7B**). *Chloromonas* sp. AsaC1 grew initially after inoculation into ampicillin-containing 3NBBM media, but failed to continue growth in the second inoculation.

## Discussion

In this study, we identified the bacterial communities co-existing in green and red algal snow samples from Mount Asahi, Japan. Compared to red snow, which persists over weeks on snow surfaces, green snow is much more difficult to find as it occurs for a shorter period of time during the snow algae life cycle (Lutz et al., 2014). Additionally, the green snow on Mount Asahi was very pale in color compared to the red snow, making it challenging to detect. In addition to the two sampling dates used for this study (Sampling day 1: June 15th, 2016; Sampling day 2: June 22nd, 2016), we also returned to the same sampling area on July 6th and observed that the red snow was still abundantly present, but were unable to detect any green snow.

The microbial community sequencing of 18S rRNA genes revealed that *Chlorophyceae* algae belonging to the genera *Chloromonas* and *Chlamydomonas* dominated in all samples (**Figure 3**). Psychrophilic algae in its green state are thought to be photosynthetically active and growing, which is supported by the OJIP transient chlorophyll fluorescence showing higher activity in green snow (Supplementary Figure S1). However, it is important to note that low OJIP transients do not indicate complete lack of photosynthetic activity, as there have been reports of oxygen evolution from red cysts obtained from snow (Remias et al., 2005). 18S rRNA-based analyses of red snow frequently identify *C. nivalis* and *Chlamydomonas nivalis* as dominant algae, and previous identification of *C. polyptera* cysts described the snow as orange-colored (Fujii et al., 2012; Remias et al., 2013b; Lutz et al., 2015b). As various *Chlorophyceae* species inhabit the snow, the correlation between pigment accumulation and metabolic activity and dormancy is difficult to determine. Nevertheless, the high concentrations of NH_4_^+^ and SO_4_^2-^ in green snow compared to red snow indicate a more growth-supporting environment in the *C. polyptera*-containing green snow (**Table 1**). Low levels of NO_3_^-^ and PO_4_^3-^ seen in our samples are consistent with previous reports on various colored snow samples (Spijkerman et al., 2012; Hamilton and Havig, 2017). The exact cause of snow algal blooms and the trigger for astaxanthin accumulation and cyst formation are unknown, and a prior study has found that despite low nutrient levels in the snow, the cells are not nutrient-limited (Spijkerman et al., 2012). As bacteria are known to more efficiently take up phosphorus, they may be a key factor in understanding nutrient supply to the algae in these conditions (Jansson, 1988). In the snow from Mount Asahi analyzed in this study, the DOC detected in the snow samples (**Table 1**) could support growth of other heterotrophic organisms.

An abundance of non-algal eukaryotes and bacteria could be detected from the green and red snow samples collected from Mount Asahi. The profile of non-algal eukaryotic phyla detected most abundantly belong to members of the phyla *Basidiomycota*, *Cercozoa*, and *Chytridiomycota*. Members of the phyla *Basidiomycota* and Chytridiomycota have also been reported to be prominent in high elevation soils, Antarctic snow, and arctic sea ice and sediment (Freeman et al., 2009; Antony et al., 2016; Hassett and Gradinger, 2016). *Cercozoa* species were also detected in arctic freshwater lakes (Charvet et al., 2014).

For the 16S rRNA gene fragment sequencing, members of the phyla *Proteobacteria* (subphylum *Betaproteobacteria*) were the most abundant in all samples. No single species appears to be prevalent from the community sequencing data (Supplementary Table S3). Numerous members of *Betaproteobacteria* may be efficient in metabolizing algal organic exudates, which are often low molecular weight organic compounds (Hellebust, 1980). There are previous reports that *Betaproteobacteria* were the most abundant bacteria in a variety of xenic algal cultures, and that many of these interactions were specialized, for example, a specific betaproteobacterium *Limnohabitans* was found to be especially well-suited for utilizing carbon sources from a particular type of alga (Simek et al., 2011). *Bacteroidetes* were also abundantly found in red snow samples, but were detected at markedly (over 100-fold) less frequency in the green snow sample. As several different algal species were detected in a limited number of samples, whether there are specific species to species interactions between bacterial strains and algae in the environment remains to be determined.

Our results at the phylum and subphylum levels were consistent with previous findings of snow from Japan, Arctic and Antarctic regions: *Betaproteobacteria* dominated in microbial communities associated with green or early season snow and members of *Bacteroidetes* were found most frequently in red snow from snowfields in Svalbard and Arctic Sweden (Segawa et al., 2005; Lutz et al., 2017). Another study looking at colored snow from snowfields in the Pacific Northwest also found members of *Betaproteobacteria* and *Bacteroidetes*, particularly *Sphingobacteria*, to be highly abundant (Hamilton and Havig, 2017). Along these lines, in a separate study, *Betaproteobacteria* were more abundant in Icelandic glacial snow samples during the early melt season (June), a period where algae show vegetative growth, and bacteria belonging to *Bacteroidetes* later in the season (August), where algae are predominantly dormant (Lutz et al., 2015a). Furthermore, a member of the *Bacteroidetes* phylum, *Hymenobacter* sp., was almost exclusively found in red snow from Antarctica, and bacteria in the class *Cytophagia*, *Saprospirae*, and *Flavobacteria* were dominant in red snow from Svalbard, and *Sphingobacteriia* were dominant in Arctic Sweden (Fujii et al., 2010; Lutz et al., 2015b, 2017).

Looking at lower taxonomic levels, findings in this study also overlap with bacteria detected at other geographical locations. Within *Betaproteobacteria*, members of the order *Burkholderiales* were prevalent from colored snow collected from Svalbard and the Pacific Northwest, as in our samples, with many highly abundant OTUs matching to *Polaromonas* sp., which was also detected in our samples (Supplementary Table S3), (Lutz et al., 2015b; Hamilton and Havig, 2017). Additionally, within the phylum *Bacteroidetes*, our two most highly detected OTUs had the top BLAST hit, albeit with low identity, to genus *Solitalea* (OTU3271 and OTU397), which is also the genus that matched to some of the most abundant OTUs in the Pacific Northwest snow algae community (Hamilton and Havig, 2017). The wide distribution of some of these bacteria in snow and ice environments is interesting, as it has been previously suggested that particles transported by wind can distribute bacteria across long distances (Maki et al., 2011). Although some bacteria likely benefit from the presence of algae, it is crucial to note that there are plenty of bacteria detected in snow without algae. Bacteria have been found ubiquitously in snow samples and bacterial growth has been detected on surface snow before algal bloom (Segawa et al., 2005; Maccario et al., 2015).

As mentioned above, the sequence belonging to the *Bacteroidetes* phylum in the red snow stem almost exclusively from OTU3271, in the class *Sphingobacteriia*. This OTU is present in trace levels (∼0.2%) in the green snow, but was frequently detected in the red snow samples (Supplementary Table S4). The reason for the low detection of members of *Bacteroidetes* in the green snow is currently unclear; it could be that *C. polyptera* abundantly found in green snow does not interact with species belonging to *Bacteroidetes*, and that these bacteria preferentially co-exist with algal strains only present in the red snow, such as strains related to *C. platystigma* and *Chlamydomonas nivalis*. In fact, previous research that identified red snow containing algae related to *Chlamydomonas nivalis* or *C. nivalis* had an abundance of bacteria belonging to the phylum *Bacteroidetes* (Fujii et al., 2010; Lutz et al., 2015b). Another speculation one could make is that members of *Bacteroidetes* are favored when algae are in the red-colored cyst stage. Some members of *Bacteroidetes* are known to thrive in low-nutrient environments and often have the ability to degrade high molecular weight carbons such as polysaccharides (Cottrell and Kirchman, 2000; Thomas et al., 2011; Gomez-Pereira et al., 2012; Johnson et al., 2016). Dead algae and residual cell wall material after algal excystation may provide carbon sources that allow bacteria of this phylum to grow, which is consistent with previous reports that found members of *Bacteroidetes* to surge in abundance after marine algal blooms (Pinhassi et al., 2004; Teeling et al., 2012). Furthermore, species from the *Bacteroidetes* phylum are frequently found attached to particles and genome analysis of several marine *Bacteroidetes* species have been found to carry increased genes for adhesion compared to *Proteobacteria* (DeLong et al., 1993; Fernandez-Gomez et al., 2013; Mohit et al., 2014). The surfaces of non-motile algal cysts found abundantly in red snow may be particularly a well-suited environment to support growth of *Bacteroidetes*. In the marine environment, *Sphingobacteria* have been found to be particularly well-equipped to attach to algal cells as their genome contained several surface adhesion proteins and peptidases for degradation of algal exudates (Gomez-Pereira et al., 2012).

In this study, all *Chloromonas* sp. colonies picked from plates spread with red snow samples matched to OTU189. Subsequent liquid culturing of these algal isolates in minimal media resulted in a consistent detection of *Betaproteobacteria* m1 and m2, which related closely to *Glaciimonas* and *Polaromonas*, respectively (**Figure 6**). Species belonging to *Bacteroidetes* were not detected in the laboratory cultures of *Chloromonas* sp. AsaC1-C7 strains, despite the fact that the algae were cultured from the red snow samples A1 and B2 that abundantly contained *Bacteroidetes* (**Figure 4**). The reason for this may be that the laboratory culture conditions used for growing this alga were unfavorable for members of the *Bacteroidetes* present in the sample and that it was out-competed by other bacteria.

*Glaciimonas*, and *Polaromonas* have been previously identified in polar and alpine environments on glaciers, snow and soil (Irgens et al., 1996; Sizova and Panikov, 2007; Weon et al., 2008; Zhang et al., 2011; Margesin et al., 2012; Franzetti et al., 2013; Frasson et al., 2015; Gawor et al., 2016; Margesin et al., 2016; Xing et al., 2016). From our data, they were among the most abundant *Betaproteobacteria* from the sequencing data (Supplementary Table S3). The presence of these bacteria in *Chloromonas* cultures may be simply due to relatively high abundance of these strains in the snow samples in combination with capabilities of efficiently utilizing algae-derived carbon sources. Both *Glaciimonas* and *Polaromonas* have been identified in environments containing phototrophs, such as cryoconite holes (Kanzler et al., 2005; Zhang et al., 2011; Franzetti et al., 2016). However, in this case, these bacteria are not the only ones benefiting from this situation, as the presence of bacteria appears to be essential for sustained growth of *Chloromonas* sp. AsaC1 (**Figure 7**). The exact nature of this effect is currently unknown due to the *Chloromonas* cultures still being xenic. As no single genus of *Betaproteobacteria* dominated across all samples in the sequencing results, the mutualistic relationship between *Betaproteobacteria* and algae may be flexible and not limited to a particular species to species interaction. These bacteria could provide algae with essential nutrients, such as vitamins (Croft et al., 2005). Alternatively, bacteria may protect the algae from possible parasites. For instance, zoosporic fungi *Chytridiomycota*, which were abundant in the 18S rRNA gene fragment sequencing results, commonly prey on algae, and bacterial antifungal agents could suppress these parasites and provide an environment for successful algal growth (Carney and Lane, 2014). The m1 strain present in many of the *Chloromonas* isolates, related to *Glaciimonas* sp., is also closely related to *Collimonas* sp., and some species among this genus are known to produce antifungal compounds (Hakvag et al., 2009; Fritsche et al., 2014). Currently the role of bacteria present in the *Chloromonas* isolates is unknown and further experiments are necessary to elucidate their relationship.

In this study, we characterized the microbial community of green and red snow samples from an alpine snowfield on Mount Asahi, in Japan. Analysis of the microbial community of the algal snow samples revealed that *Chlorophyceae* algae of the genera *Chloromonas* and *Chlamydomonas* are the major algae present in the colored snow. Bacteria belonging to the subphylum *Betaproteobacteria* are abundant in all snow samples, and members of the *Bacteroidetes* phylum were also prevalent in the red snow samples. Additionally, the *Chloromonas* sp. AsaC1-C7 isolates, related to *C. platystigma*, originating from red snow with abundant bacteria belonging to *Bacteroidetes* phylum, were found to be growing with *Betaproteobacteria* when cultured in the laboratory. The bacterial community profile data in combination with culture experiments suggest that certain strains of *Betaproteobacteria* can efficiently grow in algae-rich psychrophilic conditions and may in turn promote algal growth.

## Author Contributions

MT, HK, and MF designed the study. MT, KU, SM, and MF carried out fieldwork. MT conducted all experiments, analyzed the data and wrote the paper with guidance and data interpretation assistance from HK and MF.

## Conflict of Interest Statement

The authors declare that the research was conducted in the absence of any commercial or financial relationships that could be construed as a potential conflict of interest.
