# Supplementary material for: Microbial Community Analysis of Colored Snow from an Alpine Snowfield in Northern Japan Reveals the Prevalence of Betaproteobacteria with Snow Algae
Source: Front Microbiol. 2017 Aug 7;8:1481. doi: 10.3389/fmicb.2017.01481 (PMC5545588; doi:10.3389/fmicb.2017.01481)
Supplement: Supplementary file 1 [file Table_1.DOCX]

**Table S1.** Percent abundance and BLAST top hit for *Chloromonas* sp. and *Chlamydomonas* sp. OTUs

| Seq # | % of total *Chloromonas/Chlamydomonas* reads | | | | BLAST Top Hit (NCBI Accession Number) | BLAST  % Identity |
| --- | --- | --- | --- | --- | --- | --- |
|  | Red A1 | Red A2 | Red B2 | Green B2 |  |  |
| OTU44 | 46 | 14 | 9 | 100 | *Chloromonas polyptera* (JQ790556) | 100 |
| OTU189 | 13 | 31 | 56 | 0 | *Chloromonas platystigma* (AF514401) | 99 |
| OTU41 | 9 | 11 | 4 | 0 | *Chloromonas* *tenuis* (AB906347) | 98 |
| OTU937 | 2 | 20 | 0 | 0 | *Chloromonas nivalis* (AF514409) | 96 |
| OTU187 | 10 | 0 | 3 | 0 | *Chloromonas chenangoensis* (AB734113) | 98 |
| OTU222 | 20 | 24 | 28 | 0 | *Chlamydomonas nivalis* (JQ790560) | 100 |

**Table S2**. Percent abundance and BLAST top hit for prominent non-algal eukaryotes

| Seq # | % of total of most abundant non-algae 18S rRNA reads | | | | BLAST Top Hit (NCBI Accession Number) | BLAST  % Identity |
| --- | --- | --- | --- | --- | --- | --- |
|  | Red A1 | Red A2 | Red B2 | Green B2 |  |  |
| OTU43 | 23 | 49 | 22 | 30 | *Phenoliferia psychrophila* (KJ708383) | 100 |
| OTU156 | 28 | 15 | 23 | 39 | *Cercozoa* sp.  (EU734845) | 93 |
| OTU93 | 4 | 8 | 10 | 3 | *Cercozoa* sp.  (EU709158) | 93 |
| OTU557 | 6 | 6 | 9 | 1 | *Oberwinklerozyma straminea* (KJ708367) | 99 |
| Other | 39 | 22 | 36 | 27 | NA | NA |

**Table S3.** Percent abundance and BLAST top hit for *Betaproteobacteria* OTUs

| Seq # | % of total *Betaproteobacteria* reads | | | | BLAST Top Hit (NCBI Accession Number) | BLAST  % Identity |
| --- | --- | --- | --- | --- | --- | --- |
|  | Red A1 | Red A2 | Red B2 | Green B2 |  |  |
| OTU3276 | 56 | 62 | 4 | 32 | *Actimicrobium* sp. (AB991689) | 99 |
| OTU4031 | 16 | 9 | 46 | 11 | *Herminiimonas* sp. (JX983165) | 98 |
| OTU1414 | 1 | 2 | 34 | 4 | *Glaciimonas* sp.  (AB991649) | 99 |
| OTU1284 | 15 | 6 | 4 | 28 | *Aquaspirillum arcticum* (NR_040898) | 98 |
| OTU312 | 6 | 11 | 1 | 0 | *Polaromonas jejuensis* (KY302289) | 99 |
| OTU3245 | 0 | 0 | 0 | 19 | *Aquaspirillum arcticum* (NR_040898) | 99 |

**Table S4.** Percent abundance and BLAST top hit for *Sphingobacteriia* OTUs

| Seq # | % of *Sphingobacteriia* reads | | | | BLAST Top Hit (NCBI Accession Number) | BLAST  % Identity |
| --- | --- | --- | --- | --- | --- | --- |
|  | Red A1 | Red A2 | Red B2 | Green B2 |  |  |
| OTU3271 | 88 | 97 | 79 | 34 | *Solitalea koreensis* (NR_044568) | 91 |
| OTU397 | 10 | 0 | 12 | 4 | *Solitalea koreensis* (NR_044568) | 89 |
| Other | 2 | 3 | 9 | 62 | NA | NA |
