# Supplementary material for: Microbial Community Analysis of Colored Snow from an Alpine Snowfield in Northern Japan Reveals the Prevalence of Betaproteobacteria with Snow Algae
Source: Front Microbiol. 2017 Aug 7;8:1481. doi: 10.3389/fmicb.2017.01481 (PMC5545588; doi:10.3389/fmicb.2017.01481)
Supplement: Supplementary file 2 [file Image_1.PDF]

Figure S1

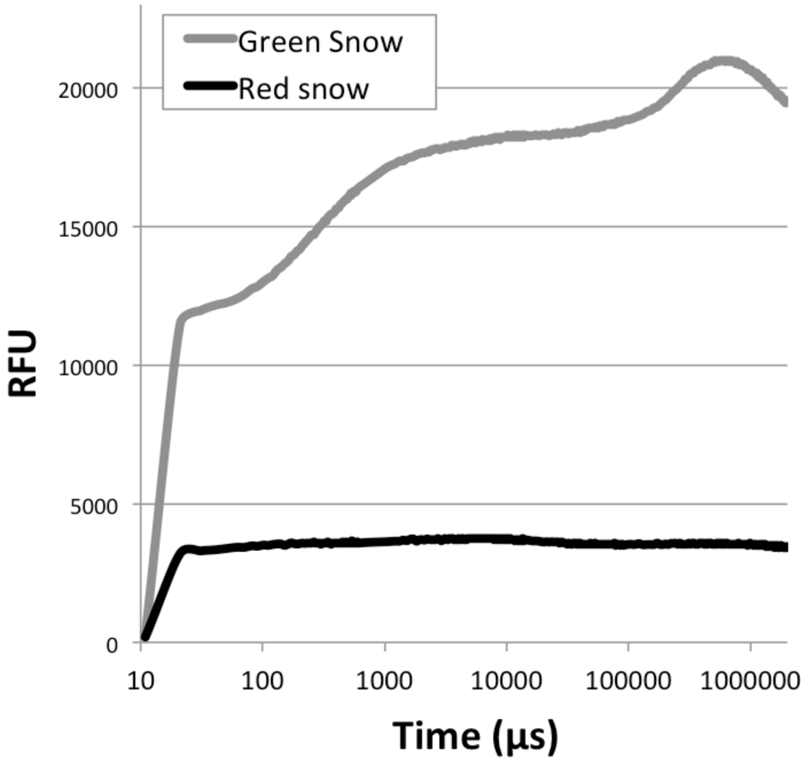

Figure S1. OJIP chlorophyll fluorescence trace of red snow B2 and green snow B2. RFU, relative fluorescence units.
