# Supplementary material for: Microbial Community Analysis of Colored Snow from an Alpine Snowfield in Northern Japan Reveals the Prevalence of Betaproteobacteria with Snow Algae
Source: Front Microbiol. 2017 Aug 7;8:1481. doi: 10.3389/fmicb.2017.01481 (PMC5545588; doi:10.3389/fmicb.2017.01481)
Supplement: Supplementary file 3 [file Image_2.PDF]

Figure S2

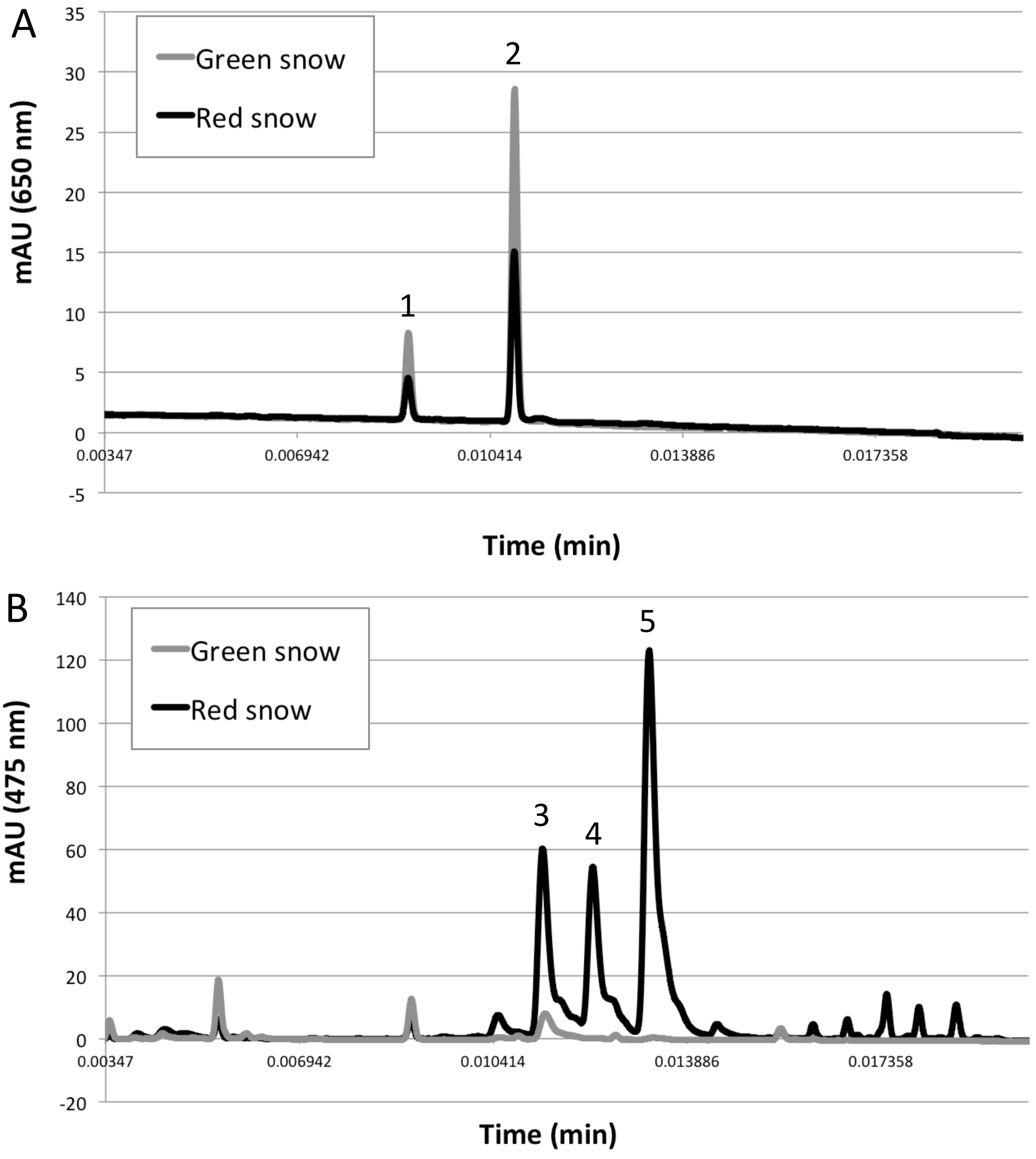

Figure S2. HPLC analysis of pigments extracted from red snow B2 and green snow B2. A. Absorption at 650 nm shows two peaks corresponding to chlorophyll b (peak 1) and chlorophyll a (peak 2). B. Absorption at 475 nm shows three prominent peaks (peaks 3-4) from the red snow, corresponding to astaxanthin esters. mAU, milli-absorbance unit
